# *In situ* patch-clamp recordings from Merkel cells in rat whisker hair follicles, an experimental protocol for studying tactile transduction in tactile-end organs

**DOI:** 10.1186/s12990-015-0022-5

**Published:** 2015-04-25

**Authors:** Ryo Ikeda, Jennifer Ling, Myeounghoon Cha, Jianguo G Gu

**Affiliations:** Department of Anesthesiology and Perioperative Medicine, College of Medicine, University of Alabama at Birmingham, 901 19TH Street South, BMR II 210, Birmingham, AL 35294 USA; Department of Orthopaedic Surgery, Jikei University School of Medicine, 3-25-8 Nishi-Shinbashi, Minato-ku, Tokyo 105-8461 Japan

**Keywords:** Piezo2 channel, Touch, Mechanotransduction, Tactile discrimination, Merkel cells, Whisker hair follicles

## Abstract

Mammals use tactile end-organs to perform sensory tasks such as environmental exploration, social interaction, and tactile discrimination. However, cellular and molecular mechanisms underlying tactile transduction in tactile end-organs remain poorly understood. The patch-clamp recording technique may be the most valuable approach for detecting and studying tactile transduction in tactile end-organs, but it is technically challenging because tactile transduction elements in an end-organ are normally inaccessible by patch-clamp recording electrodes. Here we describe an *in situ* patch-clamp recording protocol for the study of tactile transduction in Merkel cells of rat whisker hair follicle*s*, one of the most sensitive tactile end-organs in mammals. This technique offers an opportunity to explore the identities and properties of ion channels that are involved in tactile transduction in whisker hair follicles, and it may also lend a useful tool for researchers to study other tactile end-organs. The experimental protocol describes procedures for 1) tissue dissection and whisker hair follicle preparation, 2) device setup and steps for performing patch-clamp recordings from Merkel cells in a whisker hair follicle, 3) methods of delivering mechanical stimuli, and 4) intra-follicle microinjection for receptor knockdown in whisker hair follicles. The main procedures in this protocol, from tissue preparation to whole-cell patch-clamp recordings, can be completed in a few hours.

## Introduction

The sense of touch by mammalian tactile end-organs is indispensable for environmental exploration, social interaction, tactile discrimination and other tasks in life. Four major tactile end-organs have been identified so far, including Merkel discs, Ruffini’s corpuscles, Pacinian corpuscles, and Meissner’s corpuscles [1]. In addition, lanceolate nerve endings and some free nerve endings also are involved in tactile transduction [1]. The identification of these tactile end-organs was initially based on anatomical evidence showing the presence of these specialized structures at tactile sensitive spots in mammals. For example, Merkel discs were found in the touch domes throughout the body and in finger tips as well as whisker hair follicles [2-4]. Later the application of nerve fiber recordings led to the detection of afferent nerve impulses following mechanical stimulation at tactile sensitive spots that had the tactile end-organs [5,6]. Previous studies using this classical recording technique revealed a number of important properties of the tactile end-organs. These properties include the tactile sub-modality, receptive field and response dynamic range of each tactile end-organ [7]. Merkel discs were found to be most sensitive to sustained indentation of the skin with very small receptive field, and the sustained indentation generated slowly adapting type I nerve impulses (SAI) [5,7]. The SAI responses of Merkel discs are believed to be sensory encodings essential for tactile discrimination for the texture and shape of an object [8-10]. While the recordings from afferent nerve fibers have revealed many functional aspects of the tactile end-organs, this classical electrophysiological approach is not powerful in revealing cellular and molecular insights of tactile transduction in the tactile end-organs. A recent advance in the field of tactile sensory physiology is the use of developmental biological and genetic approaches to delineate the formation and neuronal connectivity of different tactile end-organs [11,12]. However, a critical question that remains to be answered is how tactile stimuli are transduced into electric signals in the tactile end-organs.

Patch-clamp recording technique may be the most direct way of detecting and studying mechanotransduction in a cell. McCarter et al. have first described mechanically activated currents (MA) in dissociated rat sensory neuron somata by using the whole-cell patch-clamp recording method [13]. The MA currents in sensory neuron somata have been characterized in more details by a number of subsequent studies using the patch-clamp recording technique [14-18]. By combining patch-clamp recordings and molecular biology approaches, it has recently been discovered that MA currents in most sensory neuron somata are mediated by Piezo2 protein, an ion channel that opens in response to mechanical displacement of cell membranes [19]. However, it remains unclear as to what extent the mechanotransduction delineated in sensory neuron somata represents tactile transduction in tactile end-organs. In addition, MA currents detected in most previous studies were evoked by directly displacing sensory neuron membranes with a glass probe. It is unknown whether the mechanical stimulation with a glass probe well mimics tactile stimulation.

The patch-clamp recording technique may be also the most useful approach to study tactile transduction in a tactile end-organ. However, it is technically challenging because tactile transduction elements in a tactile end-organ are normally inaccessible by a patch-clamp recording electrode. Nevertheless, recently we have successfully applied patch-clamp recording technique to study tactile transduction in Merkel discs of rat whisker hair follicles, and demonstrated that Merkel cells transduce tactile stimulation via Piezo2 channels [20]. Our study, together with two other recent studies using cultured Merkel cells [21,22], have for the first time revealed cellular and molecular mechanisms underlying tactile transduction in the Merkel discs. A brief description of the methods of our study has been reported elsewhere [20]. Here we describe the technical details about the *in situ* patch-clamp recordings from Merkel cells in rat whisker hair follicles. This technique offers an opportunity to further characterize the properties of tactile transduction and encoding in Merkel discs of whisker hair follicles. It may also lend a useful technical tool for studying other tactile end-organs.

## Materials

### Animals

Animal care and use conformed to NIH guidelines for care and use of experimental animals. Experimental protocols were approved by the Institutional Animal Care and Use Committee (IACUC) at the University of Alabama at Birmingham. Unless otherwise indicated, experimental animals were Sprague Dawley rats aged 10–22 days purchased from Harlan Laboratories.

### Equipment

The following instruments were used in our experiments for preparing whisker hair follicles, performing patch-clamp recordings, delivering mechanical stimuli, and knocking down candidate tactile transducers: Dissection microscope (Olympus); Dissection scissors and Forceps; Brown-Flaming P-97 programmable pipette puller (Sutter Instrument Company, Novato, CA); Gravity-fed bath perfusion system; Microforge (World Precision Instruments, Sarasota, FL); Computer-programmable Piezoelectric actuator (E-625 LVPZT; Physik Instrumente); Thin-walled borosilicate glass tubing (inner diameter 1.12 mm, outer diameter 1.5 mm, World Precision Instruments); 35-mm culture dishes (Thermo Scientific); Syringe (50 ml); Patch-pipette fillers with solution filter (4 mm diameter, 0.2 μm pore size, World Precision Instruments); Olympus IX50 upright microscope equipped with IR-DIC and fluorescent imaging systems (Olympus); Micromanipulators and pipette holder for patch clamping (Sutter Instrument Company); Multiclamp 700A amplifier, Digidata 1322A, and pCLAMP10 software (Molecular Devices, Sunnyvale, CA); Vibration isolation table and perimeter Faraday cage (TMC, Peabody, MA); Cool SNAP™ HQ^2^ CCD camera (Photometrics, Tucson, AZ); MetaFluor Imaging System software (Molecular Devices); High-speed pressure-clamp device (ALA Scientific Instruments, Farmingdale, NY 11735); Isoflurane anesthesia machine (World Precision Instruments); Microinjection system (World Precision Instruments); Digitized stereotaxic apparatus (World Precision Instruments).

### Reagents and solution preparation

Reagents for making recording electrode internal solution and bath solution were obtained from Sigma-Aldrich (St. Louis, MO). For whole-cell voltage-clamp experiments, Cs^+^-based internal solution was used and the solution contained (in mM): 70 Cs_2_SO_4_, 0.5 CaCl_2_, 2 MgCl_2_, 5 EGTA, 5 HEPES, 5 Na_2_ATP and 0.5 GTP-TRIS salt; the pH of the solution was adjusted to 7.3 with CsOH. For whole-cell current-clamp experiments, K^+^-based internal solution was used and the solution contained (in mM): 135 K-gluconate, 5 KCl, 0.5 CaCl_2_, 2 MgCl_2_, 5 EGTA, 5 HEPES, 5 Na_2_ATP and 0.5 GTP-TRIS salt; the pH of the solution was adjusted to 7.3 with KOH. The K^+^-based internal solution for whole-cell current-clamp recordings could also be used for whole-cell voltage-clamp experiments. The recording electrode internal solutions were aliquoted (0.5 ml each tube) and stored at −20°C.

Normal Krebs solution was used as the bath solution for the perfusion of whisker hair follicle tissues during patch-clamp recording experiments. A 10X stock Krebs solution was first made and the stock solution contained (in mM): 1170 NaCl, 35 KCl, 25 CaCl_2_, 12 MgCl_2_, 12 NaH_2_PO_4_, 250 NaHCO_3_ and 110 glucose. The stock Krebs solution was diluted by 10 times with de-ionized distilled water, and the pH adjusted to 7.35 with NaOH and osmolarity adjusted to 325 mOsm with sucrose to form the final Krebs bath solution. The bath solution was saturated with 95% O_2_ and 5% CO_2_ during experiments, and the solution was only used in the same day. All patch-clamp recording experiments were performed with the temperature of Krebs bath solution at 23°C.

An enzyme solution was used to help removing tissues that covered the Merkel cell layer in a hair follicle. The enzyme solution contained 0.05% dispase II plus 0.01% collagenase. The solution was prepared freshly with Krebs bath solution and used in the same day. Quinacrine stock solution (0.3 mM) was made in de-ionized distilled water and stored at 4°C. The solution was diluted 1000 times with Krebs bath solution to the final concentration of 0.3 μM and used in the same day. Lentiviral particles that carry Piezo2 shRNAs were obtained from Santa Cruz Biotechnology, Inc.

## Eperimental procedures

### Dissect whisker hair follicles from rat whisker pads **●** TIMING ~30 min

**Figure 1:**
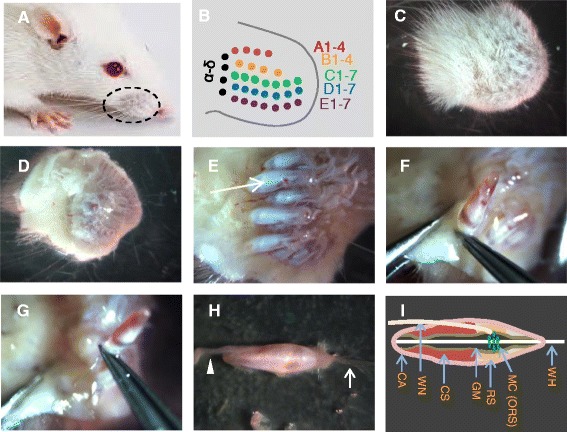
Dissection procedures for harvesting whisker hair follicles from rat whisker pads. **A)** A circle outlines the location of the whisker pad in a rat. **B)** Diagram illustrates the organization of whisker hairs in a rat whisker pad. Each whisker hair is represented by a round dot and designated according to its location in a whisker pad, e.g. A1 is the first whisker in the first row. **C)** A whisker pad was cut off from the face of a rat and placed in a dish with its hairy side up. **D-E)** Images show the tissue side (inside) of the whisker pad before **(D)** and after **(E)** the removal of fat tissues. Individual whisker hair follicles can be clearly seen after the removal of fat tissues, and one of them is indicated by an arrow. **F-G)** Pulling out whisker hair follicles. **H)** Image shows a whole whisker hair follicle with its whisker hair (arrow indicated) and whisker nerve (arrowhead indicated) after being pulled out of the whisker pad. **I)** Schematic diagram shows some main structures of a rat whisker hair follicle. CA, capsule; WN, whisker nerve; CS, cavernous sinus; GM, glassy membranes; RS, ring sinus; MC, Merkel cells; ORS, outer root sheath; WH, whisker hair shaft.

Sprague Dawley rats aged 10–22 days were chosen to make whisker hair follicle preparations for *in situ* patch-clamp recordings from Merkel cells. The reason for using postnatal animals is because their hair follicle tissues are softer so that tissue layers over Merkel cells could be removed. The removal of the tissue layers over Merkel cells is essential for patch-clamp recording electrodes to access Merkel cells and make recordings from them (see below). To dissect out whisker hair follicles, animals were first anesthetized with isoflurane and then sacrificed by decapitation. Whisker hairs in rodents are mostly located on whisker pads, the two sides of the facial regions above the upper lip and near the nose (Figure 1A). There are 5 rows of whisker hairs (designated as rows A to E) that are present on each whisker pad. In addition, 4 very large whiskers (designated as α to δ) are located at the caudal site of each whisker pad (Figure 1B) [23]. We cut off whisker pads from rats using a pair of dissecting scissors and placed them in a 35-mm petri dish that contained 2 ml ice cold L-15 medium (Figure 1C-D). Figure 1C shows the hairy side of a whisker pad; dissecting out whisker hair follicles from this site would be time-consuming. Figure 1D shows the other side of the whisker pad and this side is covered by some fat tissues. Hair follicles are embedded in the fat tissues and can be seen after removing the fat tissues using a pair of forceps (Figure 1E). Under a dissection microscope, individual whisker hair follicles and their hair shafts were carefully pulled out from whisker pads using a pair of forceps (Figure 1F-G) and placed in another 35-mm petri dish that contained 2 ml cold L-15 medium (Figure 1H). It may physically damage the cells within a whisker hair follicle if a forceps is directly applied onto a hair follicle to pull it out of the skin. To avoid such a physical damage, we first used a pair of forceps to reach the hair shaft and then held the hair shaft and pulled whisker hair and its follicle together out of the whisker pad (Figure 1G-H). Figure 1I illustrates whisker hair follicle structures that are closely relevant to the protocol of the *in situ* patch-clamp recording from Merkel cells. These structures are whisker follicle capsule, whisker nerve, cavernous sinus; glassy membranes, ring sinus, Merkel cells and outer root sheath, and whisker hair shaft. Detailed structures of whisker hair follicles in different species have been described elsewhere [23]. As illustrated in Figure 1I, Merkel cells are mainly located in the enlargement part of a whisker hair follicle. They reside in the outer root sheath (ORS), a cell layer underneath the glassy membranes in a whisker hair follicle (Figure 1I).

### Prepare hair follicles for in situ patch-clamp recordings from Merkel cells **●** TIMING ~45 min

**Figure 2:**
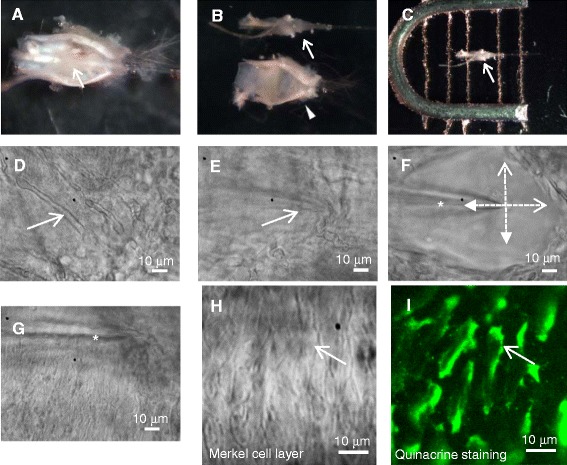
Preparation of whisker hair follicles for the *in situ* patch-clamp recordings from Merkel cells. **A)** Image shows a whisker hair follicle with its capsule cut open using an eye scissors. The arrow indicates the core of the whisker hair follicle (Follicle core). **B)** The follicle core (arrow indicated) is separated from the capsule (arrowhead indicated). **C)** The follicle core is affixed in a recording chamber by a tissue anchor. The arrow indicates the enlargement part of the follicle core. **D)** Image shows the surface of the enlargement part of the follicle core viewed under a 40X objective. Some whisker afferent nerve fibers can be observed in this region (arrow indicated). **E)** A glass electrode (arrow indicated) is used to break through the tissue layers and its tip reaches the glassy membranes in the follicle core. **F-G)** The electrode (star indicated) is moved forward, backward, left and right (indicated by dashed lines) to push off the tissue barrier **(F)** and to expose the underneath Merkel cell layer **(G)**. **H-I)** Image shows Merkel cell layer in the whisker hair follicle preparation after vital staining with quinacrine. The image in **H** is under a bright field and in **I** under a fluorescent microscope (40X objective). The arrow in **H** and **I** indicates a Merkel cell, and in **I** the cell shows green fluorescence.

Whole whisker hair follicles with attached afferent nerve roots (Figure 1H) have been previously used to record whisker afferent SA1 (slowly adapting type 1) responses induced by hair movement [24]. However, whole whisker hair follicles are not suitable for patch-clamp recordings from Merkel cells since the capsules and other tissue layers (e.g. ring sinus tissues and glassy membranes, Figure 1I) prevent patch-clamp recording electrodes from accessing Merkel cells. It is therefore necessary to remove capsules and other tissue layers before performing patch-clamp recordings. To do so, under a dissection microscope we cut open the capsule of each whisker hair follicle with a fine eye scissors (Figure 2A), and then separated the core of the whisker hair follicle (follicle core) from its capsule by using a pair of forceps (Figure 2B). The follicle cores with their hair shafts were then affixed in a recording chamber by a tissue anchor (Figure 2C) and submerged in the Krebs baht solution. The recording chamber was mounted on the stage of the Olympus IX50 microscope that was equipped with IR-DIC and fluorescent imaging system. Under the microscope with a 40X objective, some whisker afferent nerve fibers could be seen on the surface of the follicle core (Figure 2D). In addition, cells in the ring sinus tissues could also be observed on the surface. Below the nerve fibers and ring sinus tissues, a layer of glassy membranes is present but the layer is not visible under the microscope. However, the glassy membranes could be “felt” by a patch-clamp recording electrode as if a wall was in front of Merkel cells to prevent the electrode from reaching the cells. To clean off these tissue barriers, we exposed the follicle cores to 0.05% dispase II plus 0.01% collagenase in the Krebs bath solution for 8–15 min, and the enzymes were then washed off with the Krebs bath solution. The enzymes were 100 times less concentrated than those normally used for the dissociation of sensory ganglion neurons, and the digestion time was 4 times shorter than that for the neuron dissociation. Over-digestion with high concentrations of enzymes and/or longer digestion time should be avoided because it could cause Merkel cell swelling. After the enzyme treatment, the tissue layers that covered Merkel cells were pushed off mechanically (Figure 2E-J). This was performed under a 40X objective and by using a glass electrode as a tissue mover. The glass electrode, controlled by a micromanipulator, was first inserted into the tissues with its tip reaching the glassy membrane layer in the enlargement part of the follicle core (Figure 2E). The tissues including glassy membranes were then pushed away by repeatedly moving the glass electrode in different directions (Figure 2F). Figure 2G shows the Merkel cell layer after the cleaning off the other tissue layers. A good whisker hair follicle preparation would be that after the above procedures Merkel cells remain to be in elongated shape similar to those in intact whisker hair follicles.

### Label Merkel cells and pre-identify them for in situ patch-clamp recordings **●** TIMING ~20 min

Although Merkel cells are mainly located in the outer root sheath in the enlargement part of a whisker hair follicle, many other cells including keratinocytes are also present in this region and intermingled with Merkel cells. When observed under microscope with a 40X objective in the bright field, Merkel cells and other cells are not distinguishable morphologically in the whisker hair follicles (Figure 2H). Therefore, one cannot assume that a recording made from a cell in this region is from a Merkel cell. To solve this problem, we labeled Merkel cells using quinacrine, a fluorescent dye that could specifically vital-stain Merkel cells [25]. Whisker hair follicle preparations were incubated with 0.3 μM quinacrine in the Krebs bath solution for 15 min to vital-stain Merkel cells. After the staining, the preparations were continuously perfused with the Krebs bath solution at a flow rate of 1.5 ml/min. Quinacrine-labeled cells (Merkel cells) were identified (Figure 2I) using a fluorescent imaging system. The fluorescent imaging system was controlled by the MetaFluor Imaging System software to acquire images with short exposure time (~200 ms). The short exposure time is required because quinacrine fluorescent intensity in Merkel cells would drop rapidly to the undetectable level with a prolonged exposure. Observing quinacrine-labeled Merkel cells through the eye pieces of a microscope should be avoided because this would lead to the photo bleach of quinacrine fluorescence due to the prolonged UV exposure. To perform patch-clamp recordings, the acquired digital fluorescent image that had Merkel cells (Figure 2I) was first displayed on a computer monitor. In the meantime the live image for the same field was viewed under bright field to identify Merkel cells (Figure 2H), and then the identified Merkel cell was approached by a patch-clamp electrode.

### Perform in situ patch-clamp recordings from Merkel cells ● TIMING 45 min

We used a P-97 Brown-Flaming Micropipette Puller to make recording electrodes for *in situ* patch-clamp recordings from Merkel cells. Our recording electrodes usually had a resistance of ~8 MΩ, about 2 times higher than the resistance of whole-cell patch-clamp recording electrodes used for other cells. The use of high-resistant electrodes facilitates the formation of gigaohm seal between the recording electrodes and Merkel cell membranes. We found that it became difficult to form the gigaohm seal with lower resistant electrodes. Since the input resistance of Merkel cells is normally extremely high (>2 gigaohm), the use of the high-resistant electrodes would not yield intolerable voltage-clamp errors. Recording electrodes were filled with K^+^-based internal solution for both whole-cell current-clamp and whole-cell voltage-clamp experiments, and filled with Cs^+^-based internal solution for whole-cell voltage-clamp experiments.

**Figure 3:**
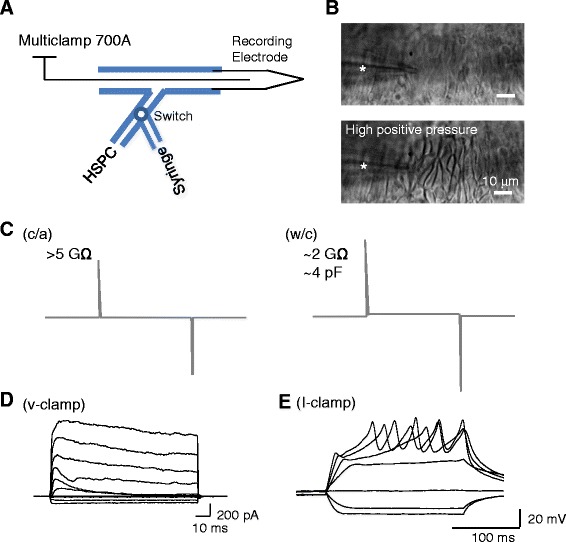
Performance of patch-clamp recordings from Merkel cells in whisker hair follicle preparation. **A)** Schematic diagram shows the setting of patch-clamp recording electrode with a dual pressure system. The recording electrode is connected to a 50-ml glass syringe and also to a high-speed pressure-clamp device (HSPC). The syringe is used to deliver a high pressure into the electrode when the electrode is approaching a targeted Merkel cell. The high pressure, generated by compressing 5 ml air in the syringe, is used to clean the surface of Merkel cell layer. The HSPC is used to deliver a lower positive pressure (~80 mmHg) during final touch of Merkel cell membranes with the recording electrode. The HSPC is also used for delivering a negative pressure during the formation of membrane seal and whole-cell configuration. **B)** Two images show the Merkel cell layer before (top) and following (bottom) the application of a high positive pressure into the recording electrode through the syringe. The recording electrode is placed near the Merkel cell layer (star indicated). Individual cells can be clearly seen following the high pressure. **C)** Illustration of membrane capacitive transient in the seal test (5 mV pulses) under the following configurations: after the formation of GΩ seal and in cell attached (c/a) configuration (left), and after breaking into whole-cell configuration (right). Vh = −70 mV. **D)** Sample traces of voltage-gated currents recorded from a Merkel cell under the whole-cell voltage-clamp mode. Vh = −70 mV. Voltage steps were applied at an increment of 20 mV from −90 mV to +50 mV. **E)** Sample traces of membrane depolarization and action potential firing in the same Merkel cell under the whole-cell current-clamp mode. Current steps were applied at an increment of 20 pA from −40 pA to +80 pA. The cell had a resting membrane potential of −50 mV.

A dual pressure system was set up to deliver positive and negative pressures into patch-clamp recording electrodes before and during the formation of membrane seal, respectively. As shown in Figure 3A, the recording electrode is connected to a 50-ml syringe to deliver a high positive pressure, and it is also connected to a high-speed pressure-clamp (HSPC) to deliver a low positive pressure as well as a low negative pressure. To start an experiment, a high positive pressure was first applied into the recording electrode by compressing ~5 ml air in the 50-ml syringe. The electrode was then lowered near the surface of the Merkel cell layer. Under the positive pressure from the electrode tip, individual cells could now be clearly observed with a 40x objective (Figure 3B bottom panel). The positive pressure was reduced to about 60–80 mmHg and maintained at this level by the HSPC device, and the recording electrode was further advanced to reach Merkel cell membranes. Once the recording electrode touched Merkel cell membranes, the positive pressure was gradually reduced and a negative pressure was applied through the HSPC until the formation of gigaohm seal (usually > 5 GΩ) between the recording electrode and the Merkel cell membranes (Figure 3C). Gigaohm seal was usually formed at the negative pressures between −50 to −100 mmHg.

We found that it was difficult to establish the whole-cell configuration by manually applying a pulse of negative pressure, a commonly used method to rupture the membrane patch for whole-cell recordings. In our experiments, a constant low negative pressure of about −45 mmHg was kept in the recording electrode by using the HSPC device. In the meantime, electric pulses (200 ms each) were applied to rupture Merkel cell membranes and establish whole-cell configuration. The magnitude of electric pulses started from 200 mV with an increment of 25 mV until breaking into whole-cell configuration. For most recordings, whole-cell configuration was established with the electric pulses of 300–450 mV. An increase of capacitive transient in membrane seal tests is an indicator for the formation of the whole-cell configuration (Figure 3C left panel), but this change could be overlooked because the capacitive transient increase was small. The reason for the small change in membrane seal tests was because Merkel cells had very high membrane input resistance (>2 GΩ) and very small whole-cell membrane capacitance (~4 pf). To further confirm the formation of whole-cell configuration, we usually examined membrane responses to a series of voltage steps in the voltage-clamp mode (Figure 3D) or to a series of current steps in the current-clamp mode (Figure 3E). In the voltage-clamp mode, prominent outward currents could be evoked in response to depolarizing voltage steps if the whole-cell configuration was established. In the current-clamp mode, a resting membrane potential of −40 mV or more negative was a good indication of the formation of whole-cell configuration. Firing regenerative action potentials in response to the injection of depolarizing currents (40 pA to 80 pA) was another clear indication of the establishment of whole-cell configuration (Figure 3E). In some recordings, abortive potentials rather than regenerative action potentials were observed. The fail of showing regenerative action potentials in a Merkel cell was most likely due to an unhealthy condition of the cell. In addition, an imperfect whole-cell mode, either due to a partial break-in or partial membrane reseal, could also be a reason for the fail of observing regenerative action potentials in Merkel cells.

### Apply mechanical stimulation and record mechanically activated currents ● TIMING 60 min

**Figure 4:**
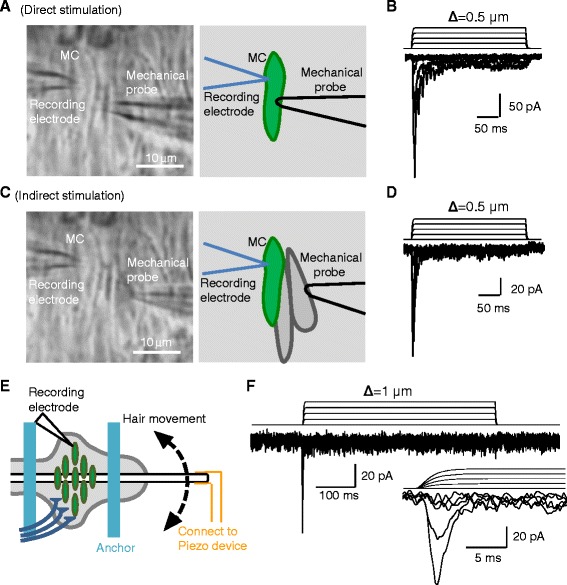
Recordings of mechanically activated currents from Merkel cells in the whisker hair follicle preparation. **A)** Image (left panel) shows the setting for the recording of Merkel cell MA currents (mechanically activated currents) evoked by direct stimulation. Diagram on right shows the outlines in left image of the recorded Merkel cell (MC, in green color), recording electrode and mechanical probe. Mechanical probe is directly applied to the recorded Merkel cell. **B)** Sample traces of Merkel cell MA currents elicited by the direct stimulation. **C)** Image (left panel) shows the setting for the recording of Merkel cell MA currents elicited by indirect stimulation. Diagram on right shows the outlines in left image of the recorded Merkel cell (MC, in green color), two non-Merkel cells (in grey color), recording electrode and mechanical probe. Mechanical probe was applied to a non-Merkel cell and mechanical force was transmitted to the recorded Merkel cell via the non-Merkel cells. **D)** Sample traces of Merkel cell MA currents elicited by the indirect stimulation. **E)** Schematic diagram illustrates the setting for the recording of Merkel cell MA currents in response to hair movement. **F)** Sample traces of Merkel cell MA currents elicited by hair movement. Inset, the same current traces at an expanded time scale.

We used three types of mechanical stimuli, direct stimulation (Figure 4A), indirect stimulation (Figure 4B), and hair movement (Figure 4C), to evoke MA currents in Merkel cells in our whisker hair follicle preparation. For the direct and indirect stimulation, we used a mechanical probe that was made by a borosilicate glass pipette. The pipette was pulled by a Brown-Flaming P-97 pipette puller to produce a tip about 1–2 μm in diameter. The tip was then smoothed by fire-polish with a microforge and the final pipette tip diameter was ~3 μm. The mechanical probe was then mounted onto a pipette holder and stably attached to a piezo device. The piezo device was computer-programmable by the pCLAMP10 software and could generate stepwise forward movement for the mechanical probe. We positioned the tip of the mechanical probe at an angle of 30 degrees to the surface of the hair follicle preparation. The distance from the tip of the mechanical probe to the targeted stimulation site was set in such a way that the tip would contact the stimulation site if the probe had one step (0.5 μm) forward movement. A slight tissue movement at the stimulation site could be observed when the first contact occurred. The tissue movement was monitored by a live image (40× objective), and the live image was acquired by a high resolution video camera and displayed on an 11-inch video monitor. For direct stimulation, the tip of the mechanical probe directly contacted the recorded Merkel cells, and a stepwise forward movement of the mechanical probe would lead to a direct membrane displacement. As shown in Figure 4B, the direct membrane displacement as small as 1 μm could evoke rapidly adapting MA currents. For indirect stimulation, the tip of the mechanical probe made a contact to the tissue at a site distant from a recorded Merkel cell (Figure 4C). In this way, the mechanical impact was transmitted across adjacent cells (~15 μm) to the recorded Merkel cell. As show in Figure 4D, the indirect stimulation also elicits the rapidly adapting MA currents.

Hair movement is a natural tactile stimulus. We applied this natural tactile stimulus to evoke MA currents from Merkel cells in our hair follicle preparation. In preparing the setup for delivering stepwise hair movement, a 26-gauge injection needle was bent into an “L” shape and was used as a holder of whisker hair. The needle was stably attached to the piezo device and a whisker hair shaft was then inserted into the tip of the needle. The hair shaft was tightly against the inside wall of the needle so that no dead space was present between hair shaft and the wall of the needle. Hair shaft was deflected by stepwise needle movements driven by the piezo device (Figure 4E). As shown in Figure 4F, hair movement elicits rapidly adapting MA currents from Merkel cells as well.

### Perform intra-follicle microinjection for knockdown of tactile transducers TIMING ~45 min

**Figure 5:**
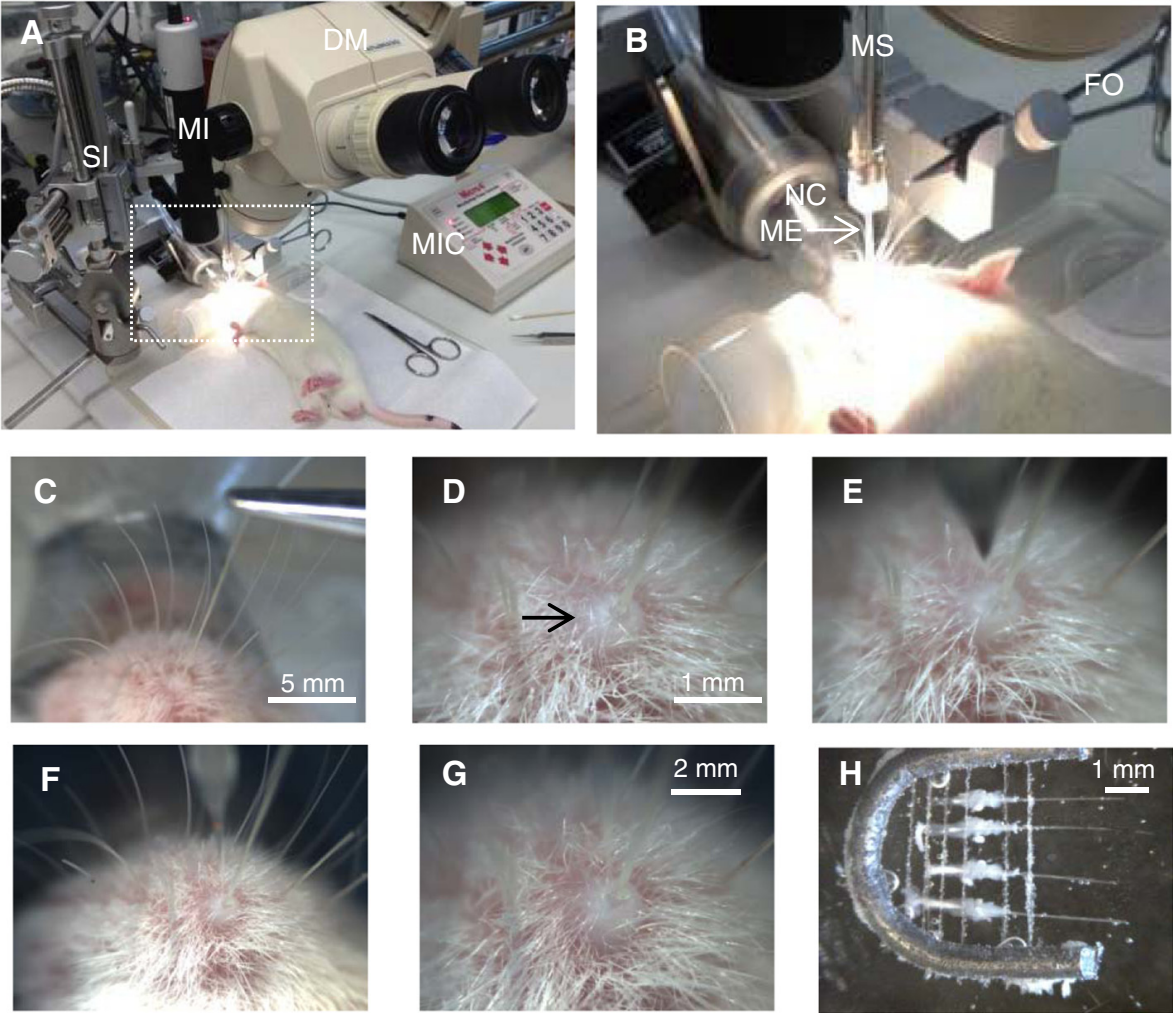
Intra-follicle microinjection of shRNA lentiviral particles for the study of mechanotransduction in whisker hair follicles. **A-B)** The experimental setting for intra-follicle microinjection. B is the enlarged image in the boxed region in A. The main instruments used for intra-follicle microinjection include a dissection microscope (DM), a stereotaxic instrument (SI), a microinjector (MI) and microinjector controller (MIC), an isoflurane anesthesia machine with its nose cone (NC), a microsyringe (MS), a forceps (FO), and a glass microelectrode (GM). **C-D)** Images show the exposure of the front part of a whisker hair follicle by gently lifting the whisker hair using a forceps. The forceps is anchored on the stereotaxic instrument as shown in **(B)**. **D** is the close-up image of the front part of the hair follicle (arrow indicated). **E)** Insertion of the glass microelectrode into the whisker hair follicle. The tip of the glass microelectrode was vertically advanced 1.5 mm deep into the whisker hair follicle. The glass microelectrode was filled with 10 μl Piezo2 shRNA lentiviral particle solution. The solution at the amount of 1 μl was microinjected into each whisker hair follicle. **F-G)** Completion of microinjection **(F)** and after withdrawal of the glass microelectrode. After the completion of the microinjection, the glass microelectrode remains within the whisker hair follicle for an additional 10 min before being withdrawn from the whisker hair follicle. **H)** Seven days after the microinjection of Piezo2 shRNA lentiviral particle solution, whisker hair follicle preparations were made for patch-clamp recordings of MA currents from Merkel cells.

To study tactile transduction and encoding in whisker hair follicles, one may want to manipulate receptor expression on Merkel cells. One way of the manipulation is to deliver shRNAs carried by lentiviral particles into whisker hair follicles. In our study, we used intra-follicle microinjection to deliver Piezo2 shRNAs carried by lentiviral particles (Piezo2 shRNAs lentiviral particles). This approach effectively knocked down Piezo2 expression on Merkel cells in whisker hair follicles [20]. The setup for the intra-follicle microinjection consisted of the following main components: an isoflurane anesthesia machine with a nose cone, a dissection microscope, a stereotaxic apparatus, and a microinjection device with a digitized controller, a glass microelectrode (Figure 5A-B). Before performing the intra-follicle microinjection, animals were anesthetized by isoflurane using the isoflurane anesthesia machine, and the anesthesia was maintained by continuous administration of isoflurane via a nose cone. A long whisker hair (D1 whisker or adjacent whisker) was chosen and a forceps was applied to the hair shaft to lift it up vertically (Figure 5B-C). The reason for choosing long whiskers is because their associated hair follicles are relatively larger and thereby microinjection is relatively easier. As shown in Figure 5C-D, after lifting a whisker hair upward, the top part of the whisker hair follicle protrudes outwardly to form a small hump on the skin surface of the whisker pad. The small hump can be clearly seen under the dissection microscope (Figure 5D). The forceps that held the whisker hair was then anchored on the stereotaxic apparatus and the tension of the lifted hair was maintain in order to continuously expose the top part of the whisker hair follicle for microinjection (Figure 5D-E). A sharp glass microelectrode with tip of ~5 μm was filled with the Piezo2 shRNAs lentiviral particle solution and it was then attached to a 25-μm microsyringe. The microsyringe was mounted on the injection holder of the stereotaxic apparatus (Figure 5A-B). Using the stereotaxic apparatus, the glass microelectrode was inserted vertically along the whisker hair into the whisker hair follicle. We usually lowered the tip of the glass microelectrode 1.5 mm deep into the whisker hair follicle, a site near the center of a large follicle (Figure 5E-F). As soon as the tip of the glass microelectrode entered a whisker hair follicle, a small amount of blood would flow from the follicle into the glass microelectrode tip. It is important to immediately start microinjection so that the blood would not aggregate in the glass microelectrode tip. We normally microinject small amount (1 μl) of the lentiviral particle solution into a whisker hair follicle because the internal space of a whisker hair follicle is small. Duration for the microinjection was usually 2.5 min and the glass microelectrode remained within the hair follicle for 10 min before being withdrawn. This avoids the possible leak of the injected solution out of whisker hair follicles. Animals were injected two times in 2 consecutive days and whisker hair follicles harvested 6–9 days after the first injection (Figure 5H). We then prepared whisker hair follicles as shown in Figure 2 for the *in situ* patch-clamp recordings from Merkel cells. As reported by our recently study, MA currents in Merkel cells of Piezo2 shRNA-injected whisker hair follicles were significantly reduced [20].

## Discussion

The protocol presented here has been successfully used by us in a recent study that demonstrates Piezo2 channel-mediated tactile transduction in Merkel cells of rat whisker hair follicles [20].This protocol can also be used for future studies to further understand 1) the transduction and encoding of tactile signals at Merkel discs, 2) the transmission of tactile signals from Merkel cells to afferent nerves, and 3) the regulation of tactile transduction, encoding and transmission at Merkel discs.

There are several advantages for the use of our *in situ* patch-clamp recording technique to study tactile transduction in whisker hair follicles. First, we used freshly harvested whisker hair follicles rather than dissociated Merkel cells. Merkel cells in our whisker hair follicle preparations remain intact and are in an *in vivo*-like condition. This avoids a potential change of tactile transduction. It has been known that receptor expression in a cell often becomes altered after the cell is dissociated and grown in culture. Previous studies have shown that dissociated Merkel cells lose their processes [26], the sites where mechanical transducers may reside in [27]. Indeed, dissociated Merkel cells failed to respond to mechanical stimulation in previous studies [28]. Interestingly, dissociated Merkel cells grown in a culture condition were reported to be mechanically sensitive in two recent studies [21,22]. Second, by using our whisker hair follicle preparations, we are able to apply several different types of mechanical stimuli to study mechanical transduction in Merkel discs of whisker hair follicles. These three stimuli are direct and indirect mechanical stimulation as well as hair movement. Importantly, since hair movement is a natural tactile stimulus, the MA currents following hair movement shown in our study thus directly illustrates tactile transduction. In dissociated Merkel cells, mechanical stimulation was achieved by direct membrane displacement with a glass probe. This stimulation paradigm does not allow one to define MA currents in Merkel cells as tactile transduction currents. Third, in our *in situ* patch-clamp recordings from Merkel cells in whisker hair follicles, we found that Merkel cells were able to fire regenerative Ca^2+^-action potentials in a slowly adapting manner [10]. On the other hand, regenerative action potentials were not demonstrated in dissociated Merkel cells [21,22,28]. The discrepancy may suggest that dissociation process and/or culture conditions alter the expression of some voltage-gated ion channels that are essential for firing regenerative action potentials in Merkel cells. The firing of Ca^2+^-action potentials in Merkel cells in response to tactile stimulation provides some insights into initial tactile encoding downstream to tactile transduction. More needs to be done using our *in situ* patch-clamp recordings to further study tactile transduction, encoding and transmission in whisker hair follicles.

In the present protocol we also describe in details the procedures for intra-follicle microinjection of Piezo2 shRNA lentiviral particles to knockdown Piezo2 channel expression in Merkel cells. As reported in our recent paper, the approach effectively knocked down Piezo2 in Merkel cells to result in a significant decrease of Merkel cell MA currents. The technical advantage of this approach is that Piezo2 shRNA lentiviral particles microinjected into a whisker hair follicle is restricted in the injected whisker hair follicle due to the insulation of its capsule. This allows highly efficient and tissue specific knockdown. This approach may also be used to knockdown other proteins of interest in Merkel cells (e.g. TRP channels) to study their potential roles in tactile transduction, encoding, and transmission in whisker hair follicles.

In addition to Merkel discs, other tactile end-organs such as Ruffini’s corpuscles, Pacinian corpuscles, and Meissner’s corpusles are involved in tactile transduction. The transduction mechanisms in these tactile organs remain to be elucidated. Our success in performing patch-clamp recordings from Merkel discs may also give a hope for the use of this technique (with modification) to study tactile transduction in these tactile end-organs.
